# Effects of hydrorelease on the mechanical properties of muscle and fascia: A study using ultrasonic shear wave elastography

**DOI:** 10.1111/cpf.70052

**Published:** 2026-02-13

**Authors:** Gakuto Nakao, Kousuke Shiwaku, Risa Adachi, Taiki Kodesho, Keigo Taniguchi, Hidenori Otsubo

**Affiliations:** ^1^ Graduate School of Health Sciences Sapporo Medical University Sapporo Japan; ^2^ Professional Post‐secondary Course (Physical Therapist), Sapporo Medical Technology, Welfare and Dentistry Professional Training College of Nishino Gakuen School Foundation Sapporo Japan; ^3^ Department of Orthopaedic Surgery, School of Medicine Sapporo Medical University Sapporo Japan; ^4^ Department of Orthopedic KKR Sapporo Medical Center Sapporo Japan; ^5^ Department of Sport Science and Research Japan Institute of Sports Sciences (JISS) Tokyo Japan; ^6^ Department of Physical Therapy, School of Health Sciences Sapporo Medical University Sapporo Japan; ^7^ Sapporo Sports Clinic Sapporo Japan

**Keywords:** fascia, hydrorelease, mechanical properties, ultrasonic shear wave elastography

## Abstract

**Purpose:**

Recent studies have shown that hydrorelease reduces gliding resistance around nerves and between fascia, alleviating pain. However, the effects of hydrorelease on the mechanical properties of muscle and fascia remain unclear. This study aimed to examine changes in the mechanical properties of muscle and fascia before and after hydrorelease.

**Methods:**

Twelve consecutive patients with ultrasound‐confirmed thickening or stacking of fascia and tenderness were included. The hydrorelease was performed under ultrasound guidance using a 25‐G, 40‐mm needle in‐plane technique, using 6 mL of saline containing 0.17% lidocaine at a concentration insufficient to produce an anaesthetic effect. The shear modulus and tenderness were measured before and after hydrorelease. The shear modulus was measured using ultrasonic shear wave elastography at two points on the muscle and fascia in the slacked and extended positions. Tenderness was evaluated for pain using the numeric rating scale. Wilcoxon signed‐rank test compared pain levels before and after hydrorelease. A two‐way analysis of variance (ANOVA) was performed for the shear modulus, with time (pre and post) and position (slacked and extended) as factors.

**Results:**

Tenderness significantly decreased after hydrorelease (*p* < 0.01). A two‐way ANOVA revealed a significant interaction (time × position) for the shear modulus in both muscle and fascia (*p* < 0.01). Post‐hoc test results indicated no change in the shear modulus in the slacked position; however, a significant decrease was observed in the extended position after hydrorelease.

**Conclusion:**

In summary, hydrorelease reduced the shear modulus of fascia and muscle during elongation, decreasing tissue stress.

## 1 INTRODUCTION

There are many definitions of myofascial pain syndrome (MPS) (Xiong et al., 2024). Common elements across these definitions are that pain arises in muscles, fascia or related soft tissues and is often accompanied by tenderness or trigger points (Cao et al., 2021). Although the detailed pathology of MPS remains unclear, several potential mechanisms have been proposed.

Previous studies have demonstrated that alterations in fascial structure, such as thickening or stacking of the fascia, are frequently observed in patients with chronic pain conditions including MPS (Langevin et al., 2009). In addition, patients with chronic low back pain exhibit approximately a 20% reduction in shear strain between fascial layers, indicating impaired fascial gliding (Langevin et al., 2011). This reduction in fascial mobility has been shown to correlate with decreased trunk flexion and extension range of motion. Collectively, these findings suggest that the thickening and stacking of the fascia in patients with MPS lead to reduced flexibility due to restricted gliding between adjacent tissues (Kimura et al., 2022; Langevin et al., 2011). Furthermore, free nerve endings associated with pain perception are densely distributed in the outer layer of the fascia and subcutaneous tissue (Tesarz et al., 2011), indicating that structural abnormalities of the fascia, such as thickening, stacking, or densification, may contribute to pain.

Although the precise pathologies of MPS remain speculative, various myofascial therapies targeting the fascia, including stretching and manual myofascial release, have been widely reported (Devantéry et al., 2023; Warneke et al., 2024). These interventions generally aim to improve the mechanical properties of the fascia and reduce pain (Devantéry et al., 2023). While static stretching has been shown to improve both muscle and fascial shear modulus and reduce pain (Warneke et al., 2024), its effects are not localized and may not sufficiently target the specific pain sites. Similarly, manual myofascial release is primarily effective for superficial tissues and has limited influence on deep soft tissues (Devantéry et al., 2023). Consequently, some patients with MPS do not experience adequate symptom relief with these conventional treatment approaches.

**FIGURE 1:**
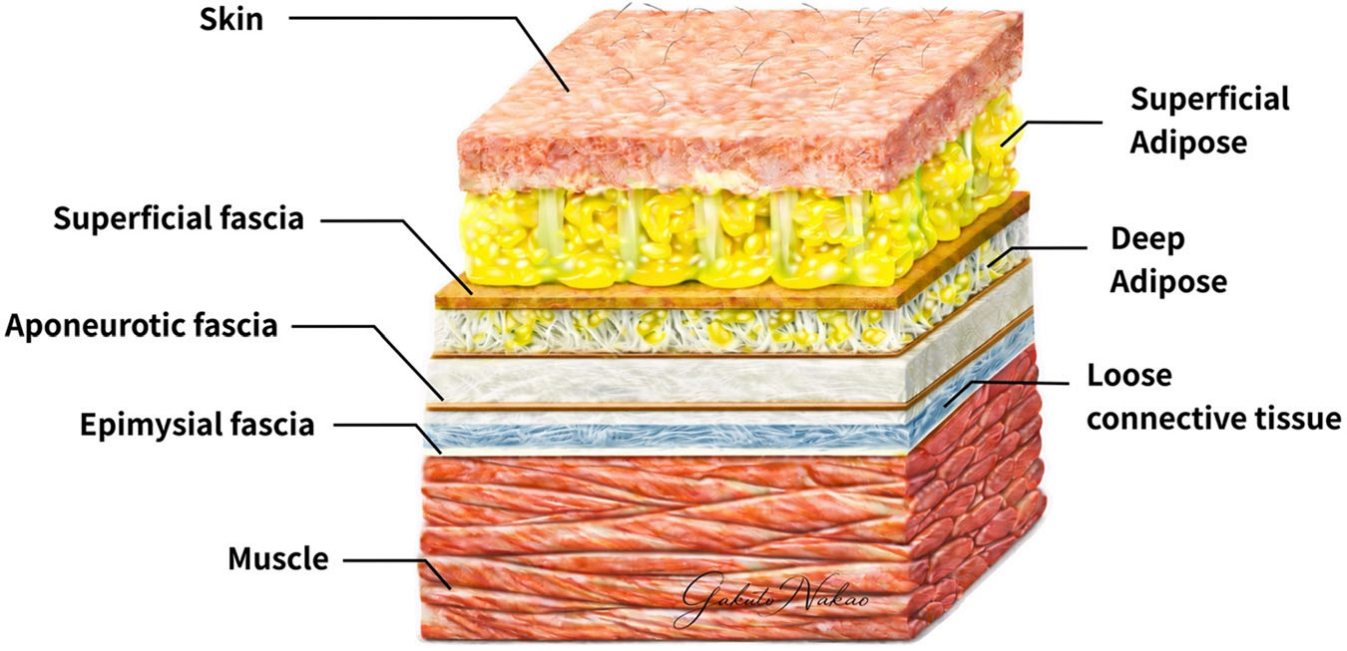
The schema of fascial layers. Schematic illustration of the layered organization of the fascia, including the superficial fascia, aponeurotic fascia, loose connective tissue, epimysial fascia, and underlying muscle.

Hydrorelease (HR), also referred to as hydrodissection, has recently been developed as a novel therapeutic approach for MPS (Evers et al., 2018; Kanamoto et al., 2021). HR involves ultrasound (US)‐guided injection of fluid into fascial tissues without inducing an anaesthetic effect and has attracted attention for its potential localized action in patients with refractory symptoms (Evers et al., 2018; Kanamoto et al., 2021; Suarez‐Ramos et al., 2023). Previous studies have reported that HR reduces gliding resistance around nerves (Evers et al., 2018) and between fascia layers (Langevin et al., 2011; Shiwaku, Otsubo, Nishikawa, et al. 2025; Shiwaku, Otsubo, Suzuki, et al. 2025; Shiwaku, Carmelo, et al. 2025), thereby alleviating pain (Kanamoto et al., 2021; Kimura et al., 2022). Understanding the anatomical organization of the fascia is essential for interpreting the mechanisms underlying HR. Fascia is composed of multiple layers, each contributing differently to musculoskeletal function and pathology (Figure 1) (Lancerotto et al., 2011; Stecco, Gesi, et al. 2013; Stecco, Tiengo, et al. 2013; Yıldızgören & Dede, 2024). Beneath the skin lies the superficial fascia, which separates the superficial and deep adipose tissues, followed by the aponeurotic fascia, characterized by collagen fibre bundles aligned along the long axis of the limb and providing high tensile resistance (Stecco et al., 2013; Stecco et al., 2016). Deeper still is the epimysial fascia, which surrounds individual muscles. Between the aponeurotic and epimysial fascia, or between adjacent epimysial layers, lies loose connective tissue rich in hyaluronic acid, which facilitates smooth fascial gliding (McCombe et al., 2001). Dysfunction of this loose connective tissue may therefore markedly impair muscle function.

Recent studies have reported that the mechanical properties of muscles in patients with MPS differ from those of healthy individuals. Shear wave elastography (SWE) has emerged as a noninvasive method for assessing the mechanical properties of soft tissues, including muscle and fascia, by quantifying the shear modulus. SWE generates shear waves within the tissue using acoustic radiation force, and the propagation velocity of these waves is used to calculate the shear modulus, which reflects the tissue's resistance to shear deformation (Hug et al., 2015). Previous research has demonstrated a close relationship between shear modulus and the passive mechanical properties of soft tissues (Kodesho et al., 2021; Koo et al., 2013; Nakao et al., 2023; Nakao, Kodesho, et al. 2025). Using SWE, muscles on the affected side in patients with MPS have been shown to exhibit a higher shear modulus on the affected side (Hao et al., 2023), with greater shear modulus observed in individuals experiencing pain (Kuo et al., 2013). Given the critical role of fascial alterations in pain development, impaired gliding, and altered mechanical properties, it is essential to identify and address these changes using appropriate therapeutic interventions. However, the effects of HR on the mechanical properties of muscle and fascia in clinical populations remain unclear. Therefore, this study aimed to investigate the changes in the mechanical properties of muscle and fascia before and after HR using ultrasonic SWE.

## 2 MATERIALS AND METHODS

### 2.1 Experimental design

Patients with myofascial pain, ultrasound‐confirmed thickening, stacking of the fascia, or densification and corresponding tenderness, who were determined to be suitable for HR were included in this study. They were evaluated using a pain scale, shear modulus in both slacked and extended positions, and flexibility. The patients were re‐evaluated 5–10 min after HR.

### 2.2 Participants

Fourteen patients were recruited for the study over 6‐day period between July 22 and 27, 2024. However, two patients were excluded due to an inability to maintain the extended position for flexibility assessment. Therefore, 12 consecutive patients with the above findings were included in the study (nine males and three females; age: 35.0 ± 21.9 years; height: 165.0 ± 8.1 cm; body weight: 62.5 ± 11.4 kg). Patients with neuromuscular disease or acute lower extremity musculoskeletal injuries were excluded. All patients received comprehensive information about the study's procedures and purpose. The study was approved by the Ethics Committee of Obihiro Kyokai Hospital, Obihiro, Japan (approval number: 2020‐26), and adhered to the requirements of the Declaration of Helsinki. All participants signed an informed consent statement prior to participation in the study. All diagnoses and inclusion decisions were made by a single board‐certified orthopaedic surgeon with over 8 years of experience in musculoskeletal ultrasound imaging.

### 2.3 US‐guided injection

**FIGURE 2:**
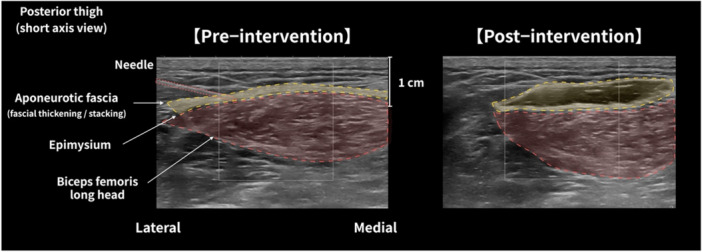
Ultrasonic short‐axis image before and after hydrorelease in the biceps femoris muscle long head. Representative short‐axis ultrasound images of the biceps femoris long head obtained before and after hydrorelease, demonstrating the anatomical relationship between the muscle and surrounding fascial layers. The yellow dashed region indicates the aponeurotic fascia exhibiting fascial thickening or stacking, identified as a hyperechoic layered structure. The red shaded region indicates the muscle belly.

In the clinic, all patients underwent ultrasound imaging, showing a hyperechoic fascial thickening (compared to other areas) and tenderness at the site of pain. After identifying hyperechoic fascial thickening, ultrasound‐guided HR was performed by injecting a total volume of 6 mL of saline containing 0.17% lidocaine, a concentration insufficient to produce an anaesthetic effect, using a 25‐G, 40‐mm needle. The needle was directed toward the area of hyperechoic changes in the fascia. Depending on the muscle, the target site was either the loose connective tissue between the aponeurotic and epimysial fascia, between the aponeurotic fascia and surrounding tissues, or both. Figure 2 shows pre‐ and post‐intervention scans of the fascia treated with HR. The intervention was performed by two orthopaedic surgeons, certified by the Japanese Orthopaedic Association, each with over 8 years of experience in performing musculoskeletal ultrasound.

### 2.4 Assessment of tenderness

Tenderness was quantified using a numerical rating scale (NRS) before and after HR, with the NRS ranging from 0 to 10, where 0 indicated no pain and 10 represented extreme pain. The same examiner conducted all evaluations.

### 2.5 Assessment of shear modulus in fascia and muscle

Shear modulus measurements were conducted in the area of fascial thickening and stacking using SWE (Aixplorer MACH 30, Hologic Supersonic Imagine, Aix‐en‐Provence, France) with a linear ultrasound transducer (5–18 MHz; Super Linear SL18‐5, Supersonic Imagine). Previous research indicates a strong linear relationship between the passive force and the shear modulus when the probe is aligned parallel to the muscle's long axis (Nakao et al., 2023), whereas no such relationship is observed when the probe is positioned transversely or along the muscle's short axis (Kodesho et al., 2021; Liu et al., 2019). Therefore, the transducer was placed parallel to the muscle's long axis.

**FIGURE 3:**
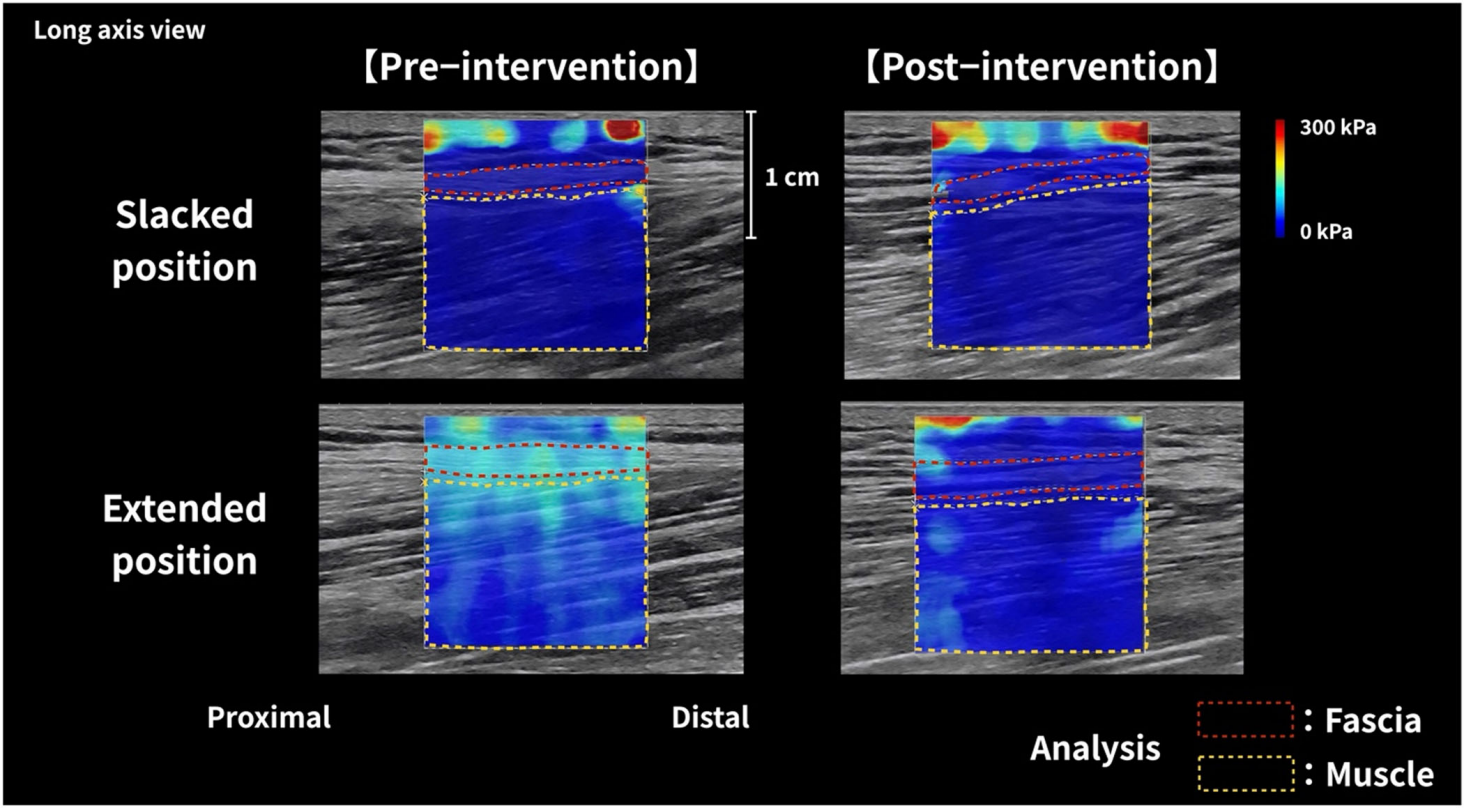
Representative shear wave elastography images before and after hydrorelease. Representative ultrasound images obtained before and after hydrorelease. The colour maps depict Young's modulus (E, kPa) as displayed by the ultrasound scanner using shear wave elastography. Hydrorelease was performed between fascial layers under ultrasound guidance, and shear wave elastography images were acquired 5–10 min after the procedure.

The shear modulus has been reported to differ between the slacked and extended positions, with higher values observed in the extended position (Nakao, Yamagata, et al. 2025; Nakao, Nara, Adachi, et al. 2025). Considering this, the effect of HR is expected to be greater in the extended position, and therefore, measurements were required in both the slacked and extended positions for each muscle. The shear modulus measurements were performed in the slacked and extended positions (Figure 3). The extension position varied for each of the seven muscles measured (rectus femoris, biceps femoris, paravertebral muscle, teres minor, gastrocnemius, flexor hallucis longus, and deltoid). For the rectus femoris, the slacked position was defined as 0° hip flexion and 0° knee extension, with the extended position set at 90° knee flexion. For the biceps femoris, the slacked position was defined as 0° straight leg raising (SLR), and the extended position was 45° SLR. For the paravertebral muscles, the slacked position was 0° trunk flexion, and the extended position was 30° trunk flexion in the lateral position. For the teres minor, the slacked position was 0° shoulder flexion, and the extended position was 90° shoulder flexion in the lateral position. For the gastrocnemius and flexor hallucis longus, the slacked position was 30° ankle plantar flexion, and the extended position was 0° ankle dorsiflexion. A TODAI‐type goniometer (Sakai Medical, Tokyo, Japan) was used to measure all joint angles.

### 2.6 Data analysis

The elastic colour map was placed in a rectangular area (20 × 20 mm) containing the thickened and stacked fascia and the underlying clear fascicle. To calculate the shear modulus, the muscle and fascia were distinguished, and the mean value of the shear modulus within the region of interest (ROI) was determined by setting as large an ROI as possible. The average muscle stiffness from two ultrasound images was used for statistical processing.

SWE generated a shear wave within soft tissues, and Young's modulus (E) in kPa was quantified based on the velocity of shear wave propagation (c). E was colour‐mapped in the ROI, calculated from each pixel using E = 3ρc^3^, where density (ρ) was assumed to be constant at 1000 kg/m^3^ for the human soft tissue (Hug et al., 2015). Although this calculation assumes tissue isotropy, muscle tissue is anisotropic and exhibits direction‐dependent mechanical properties (Hug et al., 2015). Therefore, the shear modulus was obtained by dividing the measured Young's modulus (E) by 3, assuming that biological soft tissues are nearly incompressible (Hug et al., 2015; Royer et al., 2011).

Shear modulus measurements were obtained from seven different muscles across multiple anatomical regions, each selected based on the presence of fascial thickening or stacking and corresponding tenderness. Because the primary aim of this study was to evaluate the general mechanical response of muscle and fascia to HR across multiple anatomical regions, the shear modulus data were pooled across treated muscles and analysed using a within‐subject pre–post design.

## 3 RESULTS

**FIGURE 4:**
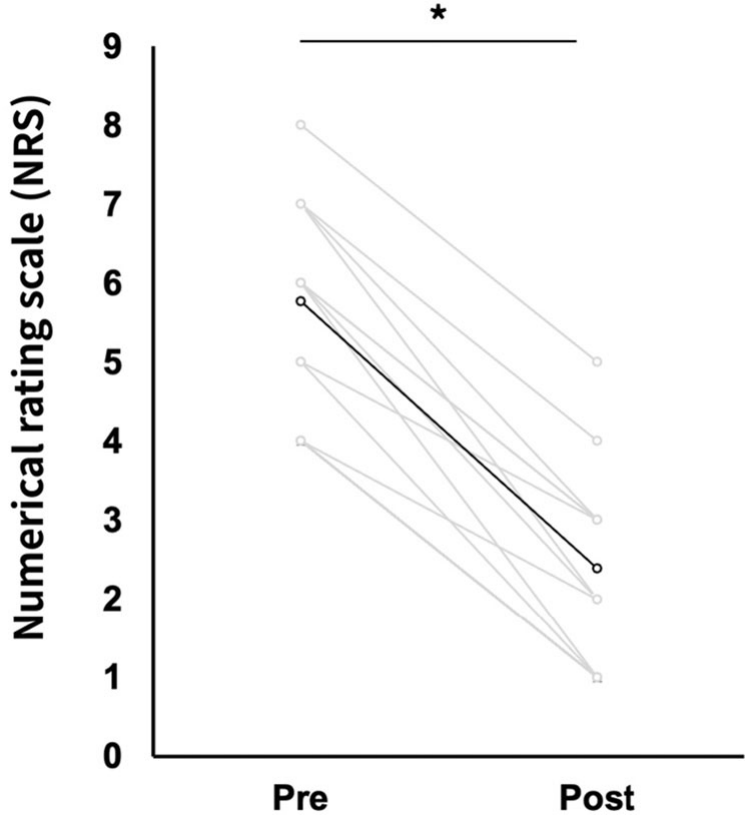
Changes in tenderness before and after hydrorelease. *p < 0.05: versus pre‐treatment. Light grey lines represent individual participant data.

**FIGURE 5:**
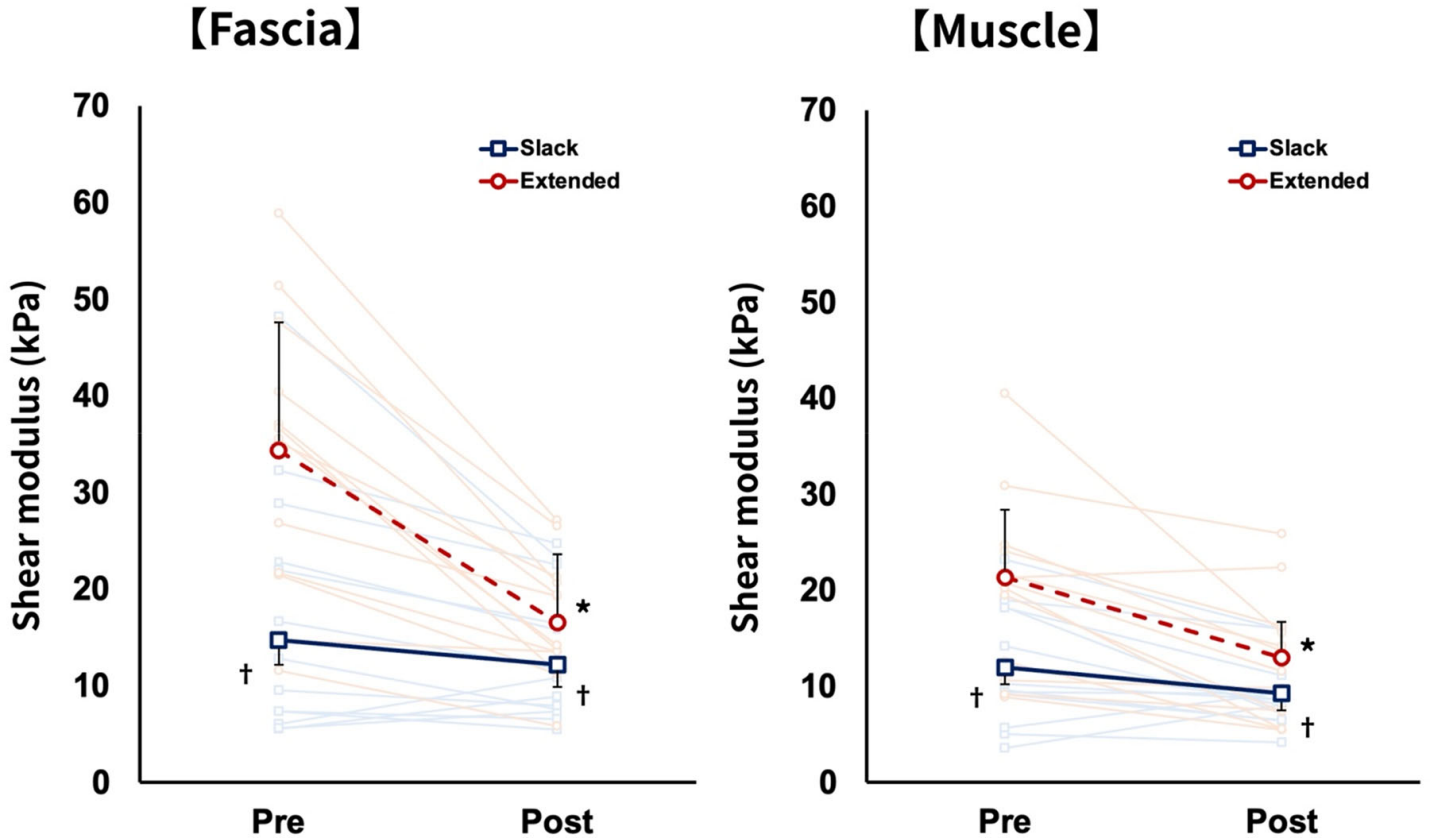
Change in shear modulus before and after hydrorelease (left: fascia, right: muscle). *p < 0.05: versus pre‐treatment. ^†^ *p* < 0.05: versus extended position. The red dashed line indicates measurements obtained in the extended position, whereas the solid blue line represents measurements obtained in the slacked position. Light red lines represent individual data acquired in the extended position, and light blue lines represent individual data acquired in the slacked position.

Given the limited number of cases for each individual muscle, data were pooled across muscles to evaluate the overall effect of HR on the shear modulus. Individual pre–post changes in the shear modulus are presented to illustrate inter‐individual variability in response to HR (Figures 4 and 5). Tenderness significantly decreased after HR compared to before HR (pre, 5.8 ± 1.25; post, 2.4 ± 1.21; *p* < 0.01) (Figure 4). For the shear modulus, two‐way ANOVA revealed a significant interaction between time and position for fascia (F_1,11_ = 29.636, *p* < 0.001, ηp^2^ = 0.729) and muscle (F_1,11_ = 8.825, *p* = 0.013, ηp^2^ = 0.445). Post‐hoc test results showed that the shear modulus of the fascia and muscle in the slacked position did not change before and after HR (fascia: pre, 14.7 ± 12.9 kPa; post, 12.1 ± 7.2 kPa, muscle: pre, 11.9 ± 6.4 kPa; post, 9.2 ± 3.4 kPa) and decreased significantly in the extended position after HR (fascia: pre, 34.3 ± 15.7 kPa; post, 16.5 ± 7.2 kPa, muscle: pre, 21.3 ± 9.8 kPa; post, 12.9 ± 6.8 kPa) (Figure 5).

## 4 DISCUSSION

### 4.1 Effects of hydrorelease on shear modulus under passive elongation

This study provides novel insights into the effects of HR on the mechanical properties of fascia and muscle, particularly in patients with MPS. Two key findings emerged from this study. Firstly, HR significantly reduced the shear modulus of both fascia and muscle in the extended position. Secondly, although the shear modulus in the slacked position remained unchanged, the pronounced decrease observed in the extended position highlights the importance of evaluating tissue properties under stretch or load, where pathological alterations are most evident. To the best of our knowledge, this is the first study to systematically assess the impact of HR on both fascia and muscle shear modulus in different positional states, offering a deeper understanding of how this intervention may reduce the shear modulus of fascia and associated symptoms.

### 4.2 Pain reduction following hydrorelease and potential underlying mechanisms

In this study, we observed a significant reduction in pain following HR, with NRS scores decreasing from 5.8 to 2.4. These results align with previous reports on pain relief after HR (Kanamoto et al., 2021; Kimura et al., 2022; Suarez‐Ramos et al., 2023). Although the precise mechanisms behind fascia‐derived pain are still under investigation, MPS‐related pain is generally thought to arise from mechanical or electrophysiological changes in the fascia and surrounding tissues, especially in areas of inflammation or ischaemia (Shiwaku, Otsubo, Nishikawa, et al. 2025; Shiwaku, Otsubo, Suzuki, et al. 2025; Shiwaku, Carmelo, et al. 2025; Stecco, Gesi, et al. 2013). Patients with MPS with thickened and stacked fascia often show increased hyaluronic acid viscosity, which may alter the mechanical properties of the fascia (Stecco et al., 2011). We speculate that one of the factors contributing to the observed thickening and high echogenicity of the fascia in patients with MPS (Stecco, Gesi, et al. 2013) may be dehydration, and the pain relief following HR might be partially attributed to the rehydration of the fascia. However, it is also possible that HR affected the surrounding nerves around the release site, resulting in a change in pain threshold. Therefore, further investigation of the mechanism of pain change is required.

### 4.3 Interpretation of shear modulus changes: Implications for muscle tension and fascial mechanics

Multiple muscles and anatomical regions were included in this study; however, all treated sites shared common pathological features, such as fascial thickening or stacking and tenderness (Figure 2). Therefore, pooling data across muscles allowed us to capture a general mechanical response to HR targeting fascial gliding dysfunction rather than muscle‐specific effects. Although the shear modulus in the slacked position remained unchanged after HR, a significant reduction was observed in the extended position for both fascia and muscle, suggesting a decrease in passive mechanical resistance during elongation (Kodesho et al., 2021; Koo et al., 2013; Nakao et al., 2023; Nakao, Kodesho, et al., 2025; Nakao, Nara, Kondo, et al. 2025). Although direct in vivo measurement of muscle or fascial tension is not feasible using current clinical imaging techniques, the comparison between slacked and extended positions at identical joint angles before and after HR provides indirect insight into changes in passive mechanical behaviour (Eby et al., 2013; Nakao, Kodesho, et al., 2025). For skeletal muscle, several cadaveric studies conducted by our group have demonstrated a strong linear relationship between shear modulus and passive muscle force under controlled elongation conditions (Kato et al., 2022; Kodesho et al., 2021; Nakao et al., 2023). From this perspective, the reduction in muscle shear modulus observed in the extended position after HR may indirectly reflect a decrease in passive muscle tension at the same level of elongation. Accordingly, when measurements are performed under standardized joint positions, SWE‐derived shear modulus may serve as a useful indirect modality for estimating changes in passive muscle tension in clinical settings, particularly under passive elongation conditions. In contrast, the interpretation of shear modulus differs for fascial tissues. Recent ex vivo evidence indicates that SWE‐derived shear modulus is not necessarily correlated with tensile properties of fascia, particularly due to tissue anisotropy and complex microstructural organization (Creze et al., 2025). Therefore, changes in fascial shear modulus observed in the present study should be interpreted primarily as alterations in local mechanical behaviour and gliding properties rather than as direct indicators of tissue tension.

### 4.4 Potential role of improved fascial gliding after hydrorelease

The reduction in the shear modulus of fascia and muscle in the extended position may be due to improved tissue gliding properties (Langevin et al., 2011; Shiwaku, Otsubo, Suzuki, et al. 2025). Myofascial pathology in the fascia of patients with MPS often leads to reduced gliding between tissues (Langevin et al., 2011), creating adhesions that increase mechanical resistance and impair movement. HR, by introducing saline into the fascial layers, may rehydrate the loose connective tissue and restore its natural viscoelasticity. This process not only facilitates smooth gliding but also alleviates mechanical stress on the peripheral nerves within the fascia, potentially mitigating perineural fascial pain, which explains why the significant reduction in stiffness was observed specifically in the extended position (Shiwaku, Otsubo, Nishikawa, et al. 2025). A previous study has shown that the shear modulus was decreased significantly by deep fascia detachment in the rectus femoris and biceps femoris muscles, which also supports the hypothesis that deep fascia affects mechanical properties (Kodesho et al., 2024; Nakao, Nara, Adachi, et al., 2025; Nakao, Nara, Kondo, et al. 2025). On the other hand, the decrease in the shear modulus may be caused by water in the tissue. Future studies should examine the effect of HR on the shear modulus in detail. These results emphasize the clinical relevance of evaluating fascial properties under dynamic or elongated conditions.

### 4.5 Study limitations and future directions

This study had several limitations. First, accurate sonographic measurement of fascial thickness remains methodologically challenging due to substantial regional variability, limited reproducibility, and difficulties in distinguishing between fascial layers, highlighting the need for standardized assessment protocols in future studies (Pirri et al., 2021; Štěpánková et al., 2024). Therefore, muscle and fascia thickness were not included as outcome measures in the present study. Second, the sample size was relatively small, including only 12 patients, which may have affected the generalizability of the findings and introduced potential statistical bias. Future studies with larger sample sizes are required to confirm these results and provide more robust conclusions. Third, this study lacked a control group, making it difficult to attribute the observed changes in pain and mechanical properties solely to HR, as no comparison to a non‐intervention group was available. Including a control group in future studies would help validate the effectiveness of HR. Fourth, potential heterogeneity among the different muscles examined should be acknowledged. Because the shear modulus data were pooled across multiple muscles with distinct anatomical and functional characteristics, muscle‐specific responses to HR may have been obscured. Although this approach was chosen to capture a general mechanical response to HR in clinical conditions characterized by fascial thickening or stacking, future studies should investigate muscle‐specific responses using stratified or region‐specific analyses. Fifth, recent ex vivo studies have demonstrated that shear wave elastography–derived measures are not necessarily correlated with tensile moduli of fascial tissues. For example, Crézé et al. reported that shear wave speed was not significantly correlated with tensile properties of the thoracolumbar fascia and erector spinae aponeurosis under controlled mechanical testing conditions (Creze et al., 2025). These findings further suggest that the shear modulus should not be interpreted as a direct measure of tissue tension, particularly in anisotropic connective tissues such as fascia. Finally, the follow‐up period was short, with assessments conducted 5–10 min after HR, leaving the long‐term effects on tissue mechanical properties, pain, and functionality unknown. Future studies should include longer follow‐up periods to assess the durability of the treatment effects.

## 5 CONCLUSION

Based on the results of this study, a significant reduction in pain levels was observed following HR, accompanied by a notable decrease in the shear modulus of both fascia and muscle in the extended position. These findings suggest that HR effectively reduces tissue mechanical properties in patients with MPS with thickening and stacking of the fascia or densification. By decreasing the shear modulus and improving tissue elongation, HR facilitated better outcomes regarding pain relief and functional mobility. These findings underscored the potential for HR to be integrated into clinical practice as a targeted intervention for patients with MPS and similar conditions characterized by increased fascial shear modulus and pain.

## AUTHOR CONTRIBUTIONS

Conceptualization: Gakuto Nakao, Kousuke Shiwaku, Taiki Kodesho, Keigo Taniguchi, Hidenori Otsubo. Methodology: Gakuto Nakao. Data Curation: Gakuto Nakao, Risa Adachi. Formal Analysis: Gakuto Nakao. Investigation: Gakuto Nakao, Risa Adachi. Writing—Original Draft: Gakuto Nakao. Writing—Review & Editing: Gakuto Nakao, Kousuke Shiwaku, Keigo Taniguchi. Supervision: Kousuke Shiwaku, Keigo Taniguchi, Hidenori Otsubo. Project Administration: Kousuke Shiwaku, Keigo Taniguchi, Hidenori Otsubo. Visualization: Gakuto Nakao. Validation: Kousuke Shiwaku, Keigo Taniguchi, Hidenori Otsubo. Resources: Keigo Taniguchi.

## CONFLICT OF INTEREST STATEMENT

The authors declare no conflicts of interest.

## Data Availability

All data generated or analyzed during this study are included in this published article.

## References

[cpf70052-bib-0001] Cao, Q.W. , Peng, B.G. , Wang, L. , Huang, Y.Q. , Jia, D.L. , Jiang, H. et al. (2021) Expert consensus on the diagnosis and treatment of myofascial pain syndrome. World Journal of Clinical Cases, 9(9), 2077–2089. 33850927 10.12998/wjcc.v9.i9.2077 PMC8017503

[cpf70052-bib-0002] Creze, M. , Lagache, A. , Duparc, F. , Broqué, M. , Persohn, S. , Slama, C. et al. (2025) Ex vivo mechanical properties of human thoracolumbar fascia and erector spinae aponeurosis under traction loading and shear wave elastography. Journal of the Mechanical Behavior of Biomedical Materials, 168, 107028. 40262430 10.1016/j.jmbbm.2025.107028

[cpf70052-bib-0003] Devantéry, K. , Morin, M. , Grimard, J. & Gaudreault, N. (2023) Effects of a myofascial technique on the stiffness and thickness of the thoracolumbar fascia and lumbar erector spinae muscles in adults with chronic low back pain: a randomized before‐and‐after experimental study. Bioengineering, 10(3), 332. 36978723 10.3390/bioengineering10030332 PMC10045407

[cpf70052-bib-0004] Eby, S.F. , Song, P. , Chen, S. , Chen, Q. , Greenleaf, J.F. & An, K.N. (2013) Validation of shear wave elastography in skeletal muscle. Journal of Biomechanics, 46(14), 2381–2387. 23953670 10.1016/j.jbiomech.2013.07.033 PMC3818126

[cpf70052-bib-0005] Evers, S. , Thoreson, A.R. , Smith, J. , Zhao, C. , Geske, J.R. & Amadio, P.C. (2018) Ultrasound‐guided hydrodissection decreases gliding resistance of the median nerve within the carpal tunnel. Muscle & Nerve, 57(1), 25–32. 28622409 10.1002/mus.25723 PMC5722677

[cpf70052-bib-0006] Hao, C.J. , Xiao, W.L. , Zhang, Q.B. & Tan, X.M. (2023) Viscoelasticity in trapezius myofascial pain syndrome: quantitative assessment using real‐time shear‐wave elastography. Annals of Medicine, 55(2), 2252442. 37676997 10.1080/07853890.2023.2252442 PMC10486288

[cpf70052-bib-0007] Hug, F. , Tucker, K. , Gennisson, J.L. , Tanter, M. & Nordez, A. (2015) Elastography for muscle biomechanics: toward the estimation of individual muscle force. Exercise and Sport Sciences Reviews, 43(3), 125–133. 25906424 10.1249/JES.0000000000000049

[cpf70052-bib-0008] Kanamoto, H. , Orita, S. , Inage, K. , Shiga, Y. , Abe, K. , Eguchi, Y. et al. (2021) Effect of ultrasound‐guided hydrorelease of the multifidus muscle on acute low back pain. Journal of Ultrasound in Medicine, 40(5), 981–987. 32840876 10.1002/jum.15473 PMC8247302

[cpf70052-bib-0009] Kato, T. , Taniguchi, K. , Kodesho, T. , Nakao, G. , Yokoyama, Y. , Saito, Y. et al. (2022) Adductor longus: an anatomical study to better understand groin pain. Clinical Anatomy, 35(7), 867–872. 35393703 10.1002/ca.23881

[cpf70052-bib-0010] Kimura, H. , Suda, M. , Kobayashi, T. , Suzuki, S. , Fukui, S. & Obata, H. (2022) Effectiveness of ultrasound‐guided fascia hydrorelease on the coracohumeral ligament in patients with global limitation of the shoulder range of motion: a pilot study. Scientific Reports, 12(1), 19782. 36396688 10.1038/s41598-022-23362-y PMC9671893

[cpf70052-bib-0011] Kodesho, T. , Kato, T. , Nakao, G. , Yokoyama, Y. , Saito, Y. , Watanabe, K. et al. (2024) Effects of superficial tissue and intermuscular connections on rectus femoris muscle shear modulus heterogeneity. Journal of Ultrasound, 27(3), 449–455. 36749499 10.1007/s40477-022-00769-x PMC11333411

[cpf70052-bib-0012] Kodesho, T. , Taniguchi, K. , Kato, T. , Mizoguchi, S. , Yamakoshi, Y. , Watanabe, K. et al. (2021) Relationship between shear elastic modulus and passive force of the human rectus femoris at multiple sites: a Thiel soft‐embalmed cadaver study. Journal of Medical Ultrasonics, 48(2), 115–121. 33576917 10.1007/s10396-020-01076-w

[cpf70052-bib-0013] Koo, T.K. , Guo, J.Y. , Cohen, J.H. & Parker, K.J. (2013) Relationship between shear elastic modulus and passive muscle force: an ex‐vivo study. Journal of Biomechanics, 46(12), 2053–2059. 23769175 10.1016/j.jbiomech.2013.05.016

[cpf70052-bib-0014] Kuo, W.H. , Jian, D.W. , Wang, T.G. & Wang, Y.C. (2013) Neck muscle stiffness quantified by sonoelastography is correlated with body mass index and chronic neck pain symptoms. Ultrasound in Medicine & Biology, 39(8), 1356–1361. 23683408 10.1016/j.ultrasmedbio.2012.11.015

[cpf70052-bib-0015] Lancerotto, L. , Stecco, C. , Macchi, V. , Porzionato, A. , Stecco, A. & De Caro, R. (2011) Layers of the abdominal wall: anatomical investigation of subcutaneous tissue and superficial fascia. Surgical and Radiologic Anatomy, 33(10), 835–842. 21212951 10.1007/s00276-010-0772-8

[cpf70052-bib-0016] Langevin, H.M. , Fox, J.R. , Koptiuch, C. , Badger, G.J. , Greenan‐ Naumann, A.C. , Bouffard, N.A. et al. (2011) Reduced thoracolumbar fascia shear strain in human chronic low back pain. BMC Musculoskeletal Disorders, 12(1), 203. 21929806 10.1186/1471-2474-12-203 PMC3189915

[cpf70052-bib-0017] Langevin, H.M. , Stevens‐Tuttle, D. , Fox, J.R. , Badger, G.J. , Bouffard, N.A. , Krag, M.H. et al. (2009) Ultrasound evidence of altered lumbar connective tissue structure in human subjects with chronic low back pain. BMC Musculoskeletal Disorders, 10, 151. 19958536 10.1186/1471-2474-10-151 PMC2796643

[cpf70052-bib-0018] Liu, J. , Qian, Z. , Wang, K. , Wu, J. , Jabran, A. , Ren, L. et al. (2019) Non‐invasive quantitative assessment of muscle force based on ultrasonic shear wave elastography. Ultrasound in Medicine & Biology, 45(2), 440–451. 30396600 10.1016/j.ultrasmedbio.2018.07.009

[cpf70052-bib-0019] McCombe, D. , Brown, T. , Slavin, J. & Morrison, W.A. (2001) The histochemical structure of the deep fascia and its structural response to surgery. Journal of Hand Surgery, 26(2), 89–97. 10.1054/jhsb.2000.0546 11281657

[cpf70052-bib-0020] Nakao, G. , Kodesho, T. , Kato, T. , Yokoyama, Y. , Saito, Y. , Ohsaki, Y. et al. (2023) Relationship between shear elastic modulus and passive muscle force in human hamstring muscles using a Thiel soft‐embalmed cadaver. Journal of Medical Ultrasonics, 50(3), 275–283. 37170041 10.1007/s10396-023-01317-8 PMC10954965

[cpf70052-bib-0021] Nakao, G. , Kodesho, T. , Yamagata, K. , Adachi, R. , Ishiyama, K. , Kozawa, K. et al. (2025) Region‐specific assessment of the mechanical properties of each hamstring muscle in human cadavers using shear wave elastography. Clinical Biomechanics, 127, 106586. 40494100 10.1016/j.clinbiomech.2025.106586

[cpf70052-bib-0022] Nakao, G. , Nara, G. , Adachi, R. , Ishiyama, K. , Kozawa, K. , Sekiguchi, K. et al. (2025) Mechanical interactions between the biceps femoris long and short heads: implications for T‐junction hamstring injuries. Clinical Physiology and Functional Imaging, 45(5), e70026. 40878013 10.1111/cpf.70026 PMC12435166

[cpf70052-bib-0023] Nakao, G. , Nara, G. , Kondo, Y. , Adachi, R. , Ishiyama, K. , Sekiguchi, K. et al. (2025) Acute effects of flossing band application on hamstring muscle mechanical properties and stretch tolerance: a randomized controlled crossover trial. European Journal of Applied Physiology. 10.1007/s00421-025-06106-4 41452474

[cpf70052-bib-0024] Nakao, G. , Yamagata, K. , Adachi, R. , Ishiyama, K. , Kozawa, K. , Watanabe, K. et al. (2025) Passive muscle tension changes in the biceps femoris long head after biceps femoris short head detachment: a human cadaver study. Journal of Biomechanics, 179, 112480. 39693787 10.1016/j.jbiomech.2024.112480

[cpf70052-bib-0025] Pirri, C. , Fede, C. , Petrelli, L. , Guidolin, D. , Fan, C. , De Caro, R. et al. (2021) An anatomical comparison of the fasciae of the thigh: a macroscopic, microscopic and ultrasound imaging study. Journal of Anatomy, 238(4), 999–1009. 33219512 10.1111/joa.13360 PMC7930759

[cpf70052-bib-0026] Royer, D. , Gennisson, J.L. , Deffieux, T. & Tanter, M. (2011) On the elasticity of transverse isotropic soft tissues (L). The Journal of the Acoustical Society of America, 129(5), 2757–2760. 21568379 10.1121/1.3559681

[cpf70052-bib-0027] Shiwaku, K. , Carmelo, P. , Otsubo, H. , Kamiya, T. , Porzionato, A. , Nakao, G. et al. (2025) Fascial ultrasound‐guided injection: where do we really inject? Cureus, 17(2), e78867. 40084316 10.7759/cureus.78867 PMC11906211

[cpf70052-bib-0028] Shiwaku, K. , Otsubo, H. , Nishikawa, D. , Itagaki, R. , Takashima, H. , Nakao, G. et al. (2025) Ultrasound‐guided fascial hydrorelease for persistent pain after hamstring injury. Journal of Functional Morphology and Kinesiology, 10(3), 318. 40843849 10.3390/jfmk10030318 PMC12371940

[cpf70052-bib-0029] Shiwaku, K. , Otsubo, H. , Suzuki, D. , Pirri, C. , Kodesyo, T. , Kamiya, T. et al. (2025) Biomechanical effects of fascial hydrorelease: a cadaveric study. BMC Musculoskeletal Disorders, 26(1), 306. 40155875 10.1186/s12891-025-08533-y PMC11951565

[cpf70052-bib-0030] Stecco, A. , Gesi, M. , Stecco, C. & Stern, R. (2013) Fascial components of the myofascial pain syndrome. Current Pain and Headache Reports, 17(8), 352. 23801005 10.1007/s11916-013-0352-9

[cpf70052-bib-0031] Stecco, A. , Stern, R. , Fantoni, I. , De Caro, R. & Stecco, C. (2016) Fascial disorders: implications for treatment. PM&R, 8(2), 161–168. 26079868 10.1016/j.pmrj.2015.06.006

[cpf70052-bib-0032] Stecco, C. , Stern, R. , Porzionato, A. , Macchi, V. , Masiero, S. , Stecco, A. et al. (2011) Hyaluronan within fascia in the etiology of myofascial pain. Surgical and Radiologic Anatomy, 33(10), 891–896. 21964857 10.1007/s00276-011-0876-9

[cpf70052-bib-0033] Stecco, C. , Tiengo, C. , Stecco, A. , Porzionato, A. , Macchi, V. , Stern, R. et al. (2013) Fascia redefined: anatomical features and technical relevance in fascial flap surgery. Surgical and Radiologic Anatomy, 35(5), 369–376. 23266871 10.1007/s00276-012-1058-0

[cpf70052-bib-0034] Suarez‐Ramos, C. , Gonzalez‐Suarez, C. , Gomez, I.N. , Gonzalez, M.K. , Co, P.H. & Llamas, J.A. (2023) Effectiveness of ultrasound guided interfascial hydrodissection with the use of saline anesthetic solution for myofascial pain syndrome of the upper trapezius: a single blind randomized controlled trial. Frontiers in Rehabilitation Sciences, 4, 1281813. 38149112 10.3389/fresc.2023.1281813 PMC10750391

[cpf70052-bib-0035] Štěpánková, T. , Quittková, A. , Čech, Z. & Machač, S. (2024) Sonographic measurement of deep fascia parameters ‐ interrater reliability. Surgical and Radiologic Anatomy, 46(9), 1481–1489. 39014213 10.1007/s00276-024-03423-9

[cpf70052-bib-0036] Tesarz, J. , Hoheisel, U. , Wiedenhöfer, B. & Mense, S. (2011) Sensory innervation of the thoracolumbar fascia in rats and humans. Neuroscience, 194, 302–308. 21839150 10.1016/j.neuroscience.2011.07.066

[cpf70052-bib-0037] Warneke, K. , Rabitsch, T. , Dobert, P. & Wilke, J. (2024) The effects of static and dynamic stretching on deep fascia stiffness: a randomized, controlled cross‐over study. European Journal of Applied Physiology, 124, 2809–2818. 38689040 10.1007/s00421-024-05495-2 PMC11365840

[cpf70052-bib-0038] Xiong, J. , Zhou, X. , Luo, X. , Gong, X. , Jiang, L. , Luo, Q. et al. (2024) Acupuncture therapy on myofascial pain syndrome: a systematic review and meta‐analysis. Frontiers in Neurology, 15, 1374542. 38765261 10.3389/fneur.2024.1374542 PMC11100351

[cpf70052-bib-0039] Yıldızgören, M.T. & Dede, B.T. (2024) The role of fascia in myofascial pain syndrome: a look at cinderella tissue. Cam and Sakura Medical Journal, 4(1), 1–8.

